# Correction: Exploring the acceptability of a community-enhanced intervention to improve decision support partnership between patients with chronic kidney disease and their family caregivers

**DOI:** 10.1371/journal.pone.0317021

**Published:** 2025-01-03

**Authors:** Shena Gazaway, Rachel Wells, John Haley, Orlando M. Gutiérrez, Tamara Nix-Parker, Isaac Martinez, Claretha Lyas, Katina Lang-Lindsey, Richard Knight, Ruth Crenshaw-Love, Allen Pazant, J. Nicholas Odom

The Data Availability statement link for this paper is incorrect. The correct link is: https://data.qdr.syr.edu/dataset.xhtml?persistentId=doi:10.5064/F6UXQABW
